# Extracellular Vesicles as a Potential Therapy for Stroke

**DOI:** 10.3390/ijms26073130

**Published:** 2025-03-28

**Authors:** Ye Sun, Gui Wan, Xinjie Bao

**Affiliations:** 1 Department of Neurosurgery, Peking Union Medical College Hospital, Chinese Academy of Medical Sciences and Peking Union Medical College, Beijing 100730, China; 15051217095@163.com (Y.S.); m15377686965@163.com (G.W.); 2 State Key Laboratory of Common Mechanism Research for Major Diseases, Beijing 100730, China

**Keywords:** stroke, extracellular vesicles, neuroprotective, ischemia, hemorrhage

## Abstract

Although thrombolytic therapy has enjoyed relative success, limitations remain, such as a narrow therapeutic window and inconsistent efficacy. Consequently, there is a pressing need to develop novel therapeutic approaches. In recent years, extracellular vesicles (EVs) have garnered increasing attention as a potential alternative to stem cell therapy. Because of their ability to cross the blood–brain barrier and exert neuroprotective effects in cerebral ischemia and hemorrhage, the exploration of EVs for clinical application in stroke treatment is expanding. EVs are characterized by high heterogeneity, with their composition closely mirroring that of their parent cells. This property enables EVs to distinguish between cerebral ischemia and hemorrhage, thus facilitating a more rapid and accurate diagnosis. Additionally, EVs can be engineered to carry specific molecules, such as miRNAs, targeting them to specific cells, potentially enhancing the therapeutic outcome and improving stroke prognosis. In this review, we will also explore the methodologies for the isolation and extraction of EVs, critically evaluating the advantages and disadvantages of various commonly employed separation techniques. Furthermore, we will briefly address current EV preservation and administration methods, providing a comprehensive overview of the state of EV-based therapies in stroke treatment.

## 1. Introduction

In a study of the Global Burden of Disease, Injuries, and Risk Factors 2021, stroke was the greatest contributor globally to the neurological burden, and its mortality rate had increased by 44.1% compared with that in 1990 [1]. Stroke include two groups: ischemic stroke, caused by the blockage of blood vessels and hemorrhagic stroke, caused by the rupture of blood vessels. At present, the most common type is acute ischemic stroke (AIS). In AIS, each minute saved in onset-to-treatment time can add an average of 4.2 days of extra healthy life, and every 20 min decrease in treatment delays can lead to a gain of an average of 3 months of disability-free life equivalency [2]. Thus, quick, precise and early diagnosis is paramount for treating patients with AIS to preserve brain issues and ultimately improve functional outcomes. Currently, relatively effective therapeutic methods simply include intravenous tissue plasminogen activator (tPA) to induce thrombolysis and endovascular thrombectomy to physically remove the blockage [3]. Furthermore, hemorrhagic stroke tends to have significant risk as the increased intracranial pressure causes a mass effect, such as midline shift and cerebral hernia, which can threaten a patient’s life. The treatment measures include pharmacological and surgical therapies to control intracranial pressure and cerebral edema. However, the use of tPA may induce or worsen the situation of hemorrhagic stroke [4]. Likewise, the therapeutic way of lowering intracranial pressure will increase the potential risk for those who suffer from ischemic stroke [5]. Therefore, medical imaging, like computed tomography (CT) or magnetic resonance imaging (MRI), is used to distinguish ischemic stroke from hemorrhagic stroke, but the examination will require a certain amount of time. Therefore, for quick diagnosis and subsequent neuroprotective therapies, extracellular vesicles (EVs) might be employed as an ideal biomarker. As discussed below, it holds true that the ideal biomarker is one that is able to pass through the BBB freely. In addition, it would be of CNS origin and correlate with distinct cell types in order to offer a window into the physiologic processes occurring in the brain during stroke. In this review, we will focus on the cutting-edge research to address current progress on EVs, providing a comprehensive overview of the state of EV-based therapies in stroke treatment.

## 2. Stroke Pathophysiology

A hallmark of ischemic stroke, interrupted blood flow, mainly caused by cerebral embolism or vascular disease, depletes the brain of oxygen and glucose [6]. First, generated reactive oxygen species result in mitochondrial dysfunction and oxidative stress injury [7]. Second, the depletion can induce electrolyte disturbance, and an imbalance of ions, such as sodium, potassium, and calcium, could contribute to the depolarization of brain tissue and the release of glutamate, which, in excess, stimulates *N*-methyl-d-aspartate receptors (NMDARs), causing neuronal excitotoxicity [8,9]. This excitotoxicity can lead to pathological changes in brain neural tissue, including cerebral edema [10], neuroinflammation [11], and cell death [12], ultimately resulting in severe neurological dysfunction and deficits [13]. These derangements, including excitotoxicity, mitochondrial dysfunction, neuroinflammation, disruption of the blood–brain barrier, and cell death, ultimately contribute to ischemic stroke [14].

During a hemorrhagic stroke, the rupture of blood vessels leads to a rapid increase in intracranial pressure, causing brain tissue deformation and mutual compression, ultimately resulting in tissue necrosis and reduced perfusion [15]. This process is driven by the release of glutamate, which activates NMDARs and induces excitotoxicity, leading to cell death [9]. Necrotic cells will activate damage-associated molecular patterns (DAMPs), which could be detected by microglia [16]. Influenced by shear force, microglia would engage ameboid form and release inflammatory factors, such as NF-κB and IL-1, triggering the recruitment of inflammatory cells, such as neutrophils and macrophages [17]. Meanwhile, the permeability of endothelial cells at the blood–brain barrier will increase to achieve the kind of recruitment, allowing many enzymes and antibodies to penetrate the parenchyma tissue, impair ion homeostasis, and amplify the oxidative stress response [18]. Astrocytes participate in ferroptosis, and increase the expression of AQP4, disrupting the water homeostasis of brain tissue [19]. Collectively, these processes result in substantial neurological damage and deficits, characteristic of hemorrhagic stroke [20].

## 3. Extracellular Vesicles

Extracellular vesicles refer to cell-derived and membrane-bound vesicles [21]. EVs can play important roles in waste removal and cell-to-cell communication via the extracellular secretory properties of all types of cells. EVs can display specific surface markers based on their original cell, which perform powerful functions in their intrinsic homing and can be taken up by cells through various endocytic pathways, such as endocytosis and micropinocytosis [22]. After uptake, EVs release their cargo, which is composed of proteins, lipids and nucleic acids [23]. According to their physical characteristics and biochemical composition, EVs can be divided into two major categories: exosomes and microvesicles.

Exosomes are the smallest types of EVs, ranging from 30–150 nm in diameter [24], and are formed by the inward budding of early endosomes, which then mature into multivesicular bodies (MVBs). MVBs are either sent to be degraded by lysosomes or fuse with plasma membranes to release their contents [25]. Exosomes contain various and sundry proteins and nucleic acids, like the family of HSP and Rabs [26]. However, they tend to include proteins associated with their endosomal secretion pathway, higher levels of glycoproteins, and proteins involved in inter-organelle crosstalk [27]. Furthermore, nuclear EVs are generated by membrane budding at the inner nuclear membrane [28]. They are passaged across the cytosol and released into the extracellular space. Nuclear EVs are rich in pre-microRNAs. Pre-microRNAs need to be processed to microRNAs to exert biological roles. Microvesicles (MVs) are larger irregularly shaped vesicles, ranging 50–1000 nm in diameter [29], and are formed by the direct outward budding of a cellular plasma membrane. MVs generally also display some of the original cell’s surface markers and contain cytosolic proteins, heat shock proteins, and integrins [30]. Apoptotic bodies with diameters larger than 500 nm are released by the outward budding of larger plasma membrane fractions as part of a cellular decomposition process in apoptotic cell death [31]. Apoptotic bodies are typically phosphatidyl-serine (PS)-decorated on their outer membrane surface, which predisposes them for clearing by phagocytes [32]. Thus, apoptotic cells may also release EVs in the exosome size that confer pro-inflammatory signals to myeloid leucocytes [33].

As said above, extracellular vesicles are heterogeneous in size, content and origin, which makes isolation difficult [34]. Therefore, to efficiently enrich EVs, different isolation methods are selected for different purposes and applications, among which ultra-centrifugation, size-based isolation techniques, polymer precipitation, and immunoaffinity capture techniques are more commonly used (Table 1).

Ultracentrifugation (UC) is currently the most widely used isolation technique and the criterion standard for extraction and separation. UC mainly harvests the re-quired components based on the size and density differences of each component in the original solution [35]. This method does not need to label EVs, which can avoid cross-contamination. However, it is not conducive to downstream analysis because of its time demand, high cost, cause of structural damage, formation of aggregates, and co-separation with lipoproteins [36]. The separation principle of size-exclusion chromatography (SEC) is that macromolecules cannot enter the gel pores and that they are eluted along the gaps between the porous gels with the mobile phase, while small molecules remain in the gel pores and are finally eluted by the mobile phase. The application of SEC is quick, easy, and low-cost. Still, they may be doped with other particles of similar size, resulting in reduced purity [37]. Immunoaffinity chromatography (IAC) is a separation and purification technology based on the specific binding of antibodies and ligands to separate desired substances from heterogeneous mixtures [38]. The binding efficiency is closely related to the biological affinity pairs, elution conditions and matrix carriers.

According to different derivations and purposes, the methods described above have various prospects. For body fluids, such as plasma and cerebrospinal fluid, and conditional cell medium, there are five purification methods commonly used to date: precipitation, membrane affinity-based separation, SEC, iodixanol gradient ultracentrifugation, and phosphatidylserine affinity isolation. For the conditioned medium, precipitation isolated the highest number of EV proteins, followed by membrane affinity-based separation. For the plasma samples, membrane affinity-based separation isolated many EV proteins and a few non-EV proteins, while precipitation isolated the most EV proteins [39]. However, it is more challenging to isolate EV from complex tis-sues, such as the brain [40]. To liberate the EVs from the extracellular matrix (ECM), the frozen or fresh tissue first must suffer an initial mechanical disruption, generally followed by enzymatic digestion to disrupt the network of glycosaminoglycans, proteoglycans, glycoproteins, and fibrous proteins that compose the ECM.

**Table 1:** The advantages and disadvantages of the main methods of isolation.

Isolation	Advantages	Disadvantages	Citation
Ultracentrifugation	Suitable for separating large-dose sample components	Low purity and recovery rate	[35,36,41]
Size-exclusion Chromatography	(1) Quick, easy, and low-cost; (2) Isolated EVs have complete structure and uniform size	Easily doped with other particles of similar size, low purity	[37]
Immunoaffinity Chromatography	Relative high purity	High-cost and troublesome	[38]

## 4. The Role of Extracellular Vesicles

The composition of EVs closely defines their biological roles. EVs abundantly contain membrane-organizing proteins, especially including tetraspanins. Tetraspanins are a family of 34 transmembrane proteins in mammals which contain four trans-membrane domains and two extracellular loops [42]. Although each tetraspanins exhibit different tissue and subcellular distributions, they are detected in nearly all cell-types as components of plasma membranes, endosomes and exosomes. Clustering with transmembrane integrins, selectins, cell adhesion molecules, cadherins and receptor proteins, tetraspanins regulate biological processes including cell adhesion, motility, proliferation and immune cell activation when stroke occurs. Associated with glycosylphosphatidylinositol (GPI)-anchored proteins and binding proteins on the outer membrane leaflet, EVs could carry various protein cargos, including cytokines, cytokine receptors, enzymes, enzyme inhibitors, ephrin, ephrin receptors, death receptor ligands and major histocompatibility complex (MHC) proteins/complexes [43,44,45]. These cargos have immunomodulatory properties and control cell proliferation, migration and guidance, as well as axonal growth. Additionally, EVs may contain RNAs, namely microRNAs, pre-microRNAs, long non-coding RNAs and mRNAs, as well as DNA [46]. What’s more, important functions of EVs have been attributed to lipids, like phosphatidic acid, phosphatidylserine (PS), and sphingolipids [31]. PS serves as signal for phagocyte removal when exposed on apoptotic cells [47], while the sphingolipids sphingomyelin and sphingosine-1 phosphate (S1P) crucially control EVs budding and release and modulate cell migration and differentiation upon target cell binding [48].

Stem cell therapy has received much attention in the past few years. However, it is undeniable that there are still many obvious safety concerns, such as the possibility of tumorigenic and immune rejection of cell transplantation [49]. EVs, as cell-free replacement therapy, have the advantage over cell therapy. Still, those derived from stem cells and other differentiated cells could show anti-inflammatory regenerative proper-ties in different tissue cells, such as the brain, lung and heart [50,51,52]. Moreover, EVs have been shown to reduce neuropathology and improve behavioral and cognitive deficits in several animal models of neurological diseases [53,54]. EVs can be enucleated by brain microvascular endothelial cells, allowing them to cross the blood–brain barrier and import foreign molecules directly into the nervous system, making EVs useful as an attractive potential treatment for neurological disorders [55,56,57]. For example, in amyotrophic lateral sclerosis, neurons can transport EV-encapsulated miR-124a to astrocytes, which elevates the glutamate transporter GLT1 by transcriptional regulation, reducing extracellular glutamate levels and reversing synaptic over-activation [58]. Additionally, in the inflamed brain, EVs are released by macro-phages, which can further transport functional NADPH oxidase NOX2 and miRNAs to neuronal axons, from which they are retrogradely carried to the perikaryon, inducing axonal regeneration via PTEN deactivation [59].

## 5. Extracellular Vesicles in Stroke Diagnosis

As previously discussed, the effectiveness of stroke treatment diminishes over time, making it critical to minimize delays in diagnosis and intervention. Currently, stroke diagnosis depends on the ability to distinguish it from conditions such as migraines and seizures, as well as to differentiate between hemorrhagic and ischemic strokes. This latter distinction typically necessitates the use of CT or MRI, both of which are time-consuming and have limitations unlikely to be resolved soon. However, recent reports have highlighted the potential of EVs as a diagnostic tool for stroke. EVs can be rapidly measured and offer promise as an acute diagnostic tool that differentiates between ischemic and hemorrhagic strokes. The discussion below further explores this potential.

Extracellular vesicles have a unique molecular profile that presents the phenotypic composition of their original cell. The current studies show that several types of cells could release EVs into the blood during stroke, including neural cells, endothelial cells, platelets, erythrocytes, and leukocytes [60,61,62]. In 2006, Simak reported a correlation between the severity of stroke and EVs originating from endothelial cells [61]. Among them, CD54/ICAM-1 positive EVs (CD105+, CD54+, CD45−) had the strongest correlation with ischemic lesion volume, while endothelial cell-derived EVs (CD105+, CD41a−, CD45−) more strongly correlated with long-term clinical outcome. Similarly, Chiva-Blanch et.al observed that in AIS patients, neural progenitor cells (CD34+, CD56+), platelets (CD61+), endothelial cells (CD146+), erythrocytes (CD235ab+), and leucocytes (CD45+) were all increased [63]. However, none of these studies could distinguish between minor–moderate and severe stroke. In short, these studies show promising results and increase the anticipation of the diagnostic potential of peripheral blood EVs in AIS.

In another study, investigators found EVs from endothelial cells (CD105+, CD106+, CD54+, or CD62e+), leucocytes (CD45+), and erythrocytes (CD235+) were elevated in patients with ICH [64]. Interestingly, Sanborn et al. not only obtained similar results but also found that the concentration of EVs from neutrophils (CD66b+), erythrocytes (CD235a+), and endothelial cells (CD146+) tended to increase [65]. These studies show a clear diagnostic potential of EVs in hemorrhagic stroke. By contrast, in one group with ischemic stroke, for example, the EVs positive for CD235a+ erythrocytes and CD105+ endothelial cells are similar to the EV population above, making it difficult to distinguish stroke subtypes.

Moreover, the elevation of EVs for several days or even months might contribute to continuous inflammatory response, edema, or a leaky blood–brain barrier, which is not relevant to acute events. Instead, these EV changes could be valuable as prognostic markers of long-term clinical outcomes or serve as treatment-monitoring biomarkers.

## 6. Extracellular Vesicles in Ischemic Stroke Treatment

**Figure 1:**
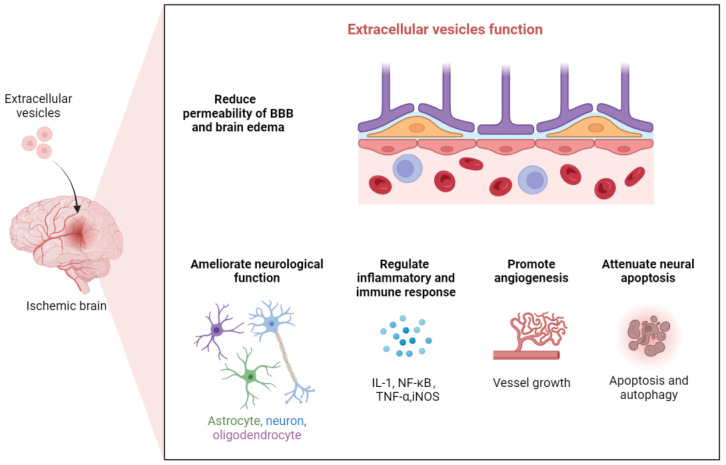
The function of extracellular vesicles in ischemic brain. Extracellular vesicles have several therapeutic functions on ischemic stroke, including ameliorating neurological function, reducing permeability of BBB and brain edema, regulating inflammatory and immune response, attenuating neural apoptosis, adjusting autophagy, and modulating angiogenesis and neurogenesis. Portrayed by Bio Render.

Extracellular vesicles have several therapeutic functions on ischemic stroke (Figure 1), including ameliorating neurological function, reducing permeability of the blood–brain barrier, brain edema, regulating inflammatory and immune response, attenuating neural apoptosis, adjusting autophagy and modulating angiogenesis and neuro-genesis [66].

The most important effect of EVs on the pathological progression of cerebral ischemia is the restoration of neurological function and the protection of brain cells. In the mice transient middle cerebral artery occlusion (t-MCAO) model, it is reported that EVs derived from stromal cells, neurons, and astrocytes, respectively, could improve nerve severity scores by reducing the production and activation of ion-calcium-binding receptor molecule (lba1)-positive microglia [67,68]. Additionally, EVs have been shown to have the same effect in large mammalian models. For example, after intravenous injection of EVs from human neural progenitor cells in a pig permanent MCAO model, it was found that the lesion volume was significantly reduced and nerve function was greatly improved [69]. In addition, EVs can play a therapeutic role in the gray matter and cortex and enhance white matter function. In the rat MCAO model, intravenous injection of EVs derived from mesenchymal stem cells can regulate the number of oligodendrocytes and astrocytes, thereby promoting axon and myelin recovery, reducing infarct size, and improving nerve function [70]. Furthermore, a combination of EVs and drugs has been proven to have a more positive effect on is-chemic stroke than using EVs alone. Rosuvastatin via oral administration combined with EVs derived from BMSCs by stereotaxic injection markedly reduced infarct volume and brain edema, and attenuated cell death in ischemic areas poststroke by down-regulating the apoptosis-associated NLRP1/3 gene and countering lipid peroxidation [71].

The second most important effect is associated with reducing blood–brain barrier permeability and cerebral edema. As mentioned above, after ischemic stroke, the structure of the blood–brain barrier will be destroyed, and the permeability of the blood–brain barrier will in-crease, resulting in leukocyte infiltration and brain edema, aggravating brain injury, and neuronal death. Intravenous administration of hypoxia-induced MSC-derived EVs can reduce the infiltration of polymorphonuclear neutrophils in the brain tissue of MCAO mice [72]. In addition, the combination of adipose stem cells and adipose-derived EVs can reduce the expression of AQP-4 and some oxidative stress molecules, such as MMP-9, TNF-α, and iNOS [73].

The significant response of brain inflammation in ischemic stroke is the activation of microglia and astrocytes and the subsequent upregulation of proinflammatory cytokines and chemokines, such as NF-κB and IL-1. Recently, some studies have found that EVs can inhibit this process. In hypoxic SHSY5Y cell models, IL-6, IL-1β, and TNF-α mRNA levels were significantly decreased after EV treatment of endothelial cells or stem cells [74]. A similar trend was observed in mouse MCAO models when administering EVs derived from astrocytes, bone marrow mesenchymal stem cells, or ADSCs [68,75,76]. In addition, EVs can inhibit the polarization of microglia toward proinflammatory (M1) or promote their anti-inflammatory (M2), polarization, thereby improving neurological outcomes [77].

Cell apoptosis occurs mainly in the penumbra during reperfusion after ischemic stroke and can further aggravate brain injury and nerve function [78]. There are primarily two apoptosis pathways: one is the endogenous pathway that activates caspa-se-3 through mitochondrial dysfunction, and the other is the exogenous pathway mediated by membrane surface apoptosis receptors that subsequently stimulate caspase-8 and ultimately directly or indirectly activate caspase-3, leading to apoptosis [79]. Mesenchymal stem cell-derived EVs can downregulate the expression of Bax and caspase-3 and the levels of IL-6, IL-1β, and TNF-α, and upregulate the expression of B-cell lym-phoma-2 (Bcl-2) and cyclin-dependent kinase 4, cyclin D1, and cyclin E, inhibiting apoptosis of astrocytes and neurons, both in OGD-induced (Oxygen and Glucose Deprivation-induced) astrocytes and in mouse MCAO models, in turn relieving injury after reperfusion [80,81]. Apart from apoptosis, autophagy plays an important role in ischemic stroke as another mechanism for cellular auto-degradation. The level of autophagy mainly depends on autophagy-related protein markers, like immunoglobulin protein (BIP) [82]. EVs derived from endothelial cells attenuated ER stress against apoptosis, regulating the crosstalk of pathways between ER stress-mediated autophagy and apoptosis after ischemic stroke [83].

After ischemia, angiogenesis can restore the metabolism of injured neurons, re-move necrotic debris, increase neuronal remodeling and promote the migration of NSCs, aggravating brain injury [84]. EVs have been shown to interfere with angiogenesis after ischemia. In the model of rat MCAO and OGD-induced endothelial cells, EVs derived from ADSCs can promote angiogenesis by evaluating endothelial cell migration and capillary formation, or by measuring vascular density in the ischemic boundary region with positive expression of endothelial cell markers vWF, the effect of which is related to endothelial microRNAs, such as miR-126 and miR-134, which may represents a novel therapeutic approach for stroke recovery [85]. Furthermore, EVs can also target neurogenesis, which is marked by Ki67 and DCX [86]. In the OGD-induced endothelial cells model, after treatment of intravenous EVs from MSCs, it suggested elevated expressions of Ki67, MOG, myelin basic protein, and synaptophysin in the infarct area of mice with subcortical ischemia, representing the enhancement of neurogenesis [87].

## 7. Extracellular Vesicles in Hemorrhagic Stroke Treatment

The treatment of cerebral hemorrhage and stroke mainly focuses on removing the hematoma and protecting the surrounding tissue of the hematoma, the latter referring to repairing the damaged nerve [88]. There are two main pathways in which EVs play a role in the treatment of cerebral hemorrhage: (1) direct action: the surface proteins on EVs recognize the receptors of target cells, inducing signal transduction, and thus transmit intercellular information; (2) indirect effect: EVs, loaded with drugs which can treat cerebral hemorrhage, fuse with target cells to deliver the loaded bioactive factors into the cell, interacting with target cell surface receptors and then achieving information transport [89]. As for the treatment of cerebral ischemia, nerve repair after cerebral hemorrhage comprises five main aspects: anti apoptosis, inflammation reduction, angiogenesis, immunomodulation, and carriers for drug delivery [90].

Neuronal cell damage caused by intracerebral hemorrhage-induced programmed cell-death is an important cause of high mortality after intracerebral hemorrhage [91]. Therefore, anti-apoptosis plays a vital role in alleviating adverse consequences and improving prognosis after intracerebral hemorrhage. Some studies have shown that in mouse models of subarachnoid hemorrhage, bone marrow mesenchymal stem cell-derived EVs are transferred to neurons via miRNA-21, promoting neuron survival and alleviating cognitive impairment after subarachnoid hemorrhage [92]. Meanwhile, miRNA-133b-modified EVs derived from MSCs can reduce neuronal apoptosis after intracerebral hemorrhage by inhibiting RhoA expression and activating ERK1/2/CREB in vitro [93]. Additionally, downregulated miRNA-206-modified EVs derived from umbilical cord MSCs, by way of targeting BDNF, mediate the TrkB/CREB signaling pathway, inhibiting apoptosis and significantly improving nerve function and brain edema, thus preventing early brain injury caused by subarachnoid hemorrhage [94].

Brain hemorrhage can cause widespread neuroinflammation, leading to expanded nerve damage. EVs can deliver miRNA, proteins, or other contents to have an anti-inflammatory effect [95]. Studies have shown that EVs derived from bone marrow mesenchymal stem cells (BMSCs) can not only reduce neuroinflammation in brain tissue after subarachnoid hemorrhage by inhibiting NF-κB and activating the AMPK pathway, acting as neuroprotective agents but also downregulate the expression of IL-1b, CD16, CD11b, and iNOS and upregulating CD206 to control the polarization of microglia toward an M2 phenotype, finally suppressing inflammation [96,97]. Another study from the same researchers found that the same type of EV can also carry miRNA 183-5p into the ICH location of rat brain, inhibiting the NLRP3 pathway by targeting PDCD4, then reducing neuroinflammation after diabetic ICH [98]. In addition, miRNA-193b3p-modified EVs can acetylate NF-κB, inhibiting HDAC3 expression and inflammation [99].

With the occurrence of cerebral hemorrhage, the vascular endothelium is destroyed, and adjacent tissues and blood vessels will also be subjected to ischemic stress, aggravating brain injury [100]. Therefore, angiogenesis plays an important role in hemorrhagic stroke prognosis. It has been reported that BMSC-derived EVs can significantly improve the angiogenesis and neurogenesis function in mice with hemorrhagic stroke, thus promoting the recovery of neurological function [101]. In addition, it has been reported that mouse brain endothelial EVs can significantly increase the axon growth of primary cortical neurons and promote endothelial capillary formation, thereby increasing axon density, myelin density, vessel density, and artery diameter, and finally improving neurocognitive function [102].

As mentioned above, EVs can be loaded with proteins or miRNA and act as mediators of intercellular communication; therefore, they are biocompatible. Transfusion of human mesenchymal stem cell EVs into immunocompetent mouse models of acute myocardial ischemia is therapeutic without significant adverse effects [103]. In addition, it has been found that MSC-EVs loaded with peroxidase can successfully cross the blood–brain barrier and improve the disease state of Parkinson’s disease [104]. However, there are no relevant reports on the treatment of cerebral hemorrhage.

## 8. Pretreatment and Clinical Trials

EV therapy has become increasingly widespread and widely used in models of central nervous system diseases in vivo [105]. Currently, EVs are routinely administered intravenously to treat experimental stroke in models in vivo. However, the ad-ministration results in a large distribution of EVs in peripheral organs (such as the liv-er, spleen, and kidneys), making EVs less likely to migrate to the brain, clear quickly delaying their effects, and causing organ toxicity [106]. Although they can penetrate the blood–brain barrier, it is difficult for EVs to migrate to the specific injury area after stroke. Some investigators have used arterial, intranasal, and direct intraventricular injections to avoid the drawbacks of intravenous injections. Intra-arterial EV injection can reduce neuroinflammation caused by focal brain injury [107]. Furthermore, direct intraventricular administration of EVs can effectively reduce microglial density and neuronal apoptosis, thereby steadily improving functional recovery in MCAO [67]. In addition, researchers have found that in the treatment of Batten’s disease, intranasal administration can better reduce astrocyte proliferation and promote neuronal recovery [108]. A study of BMSCs showed that in the rat model of MCAO, the intra-arterial pathway showed the greatest degree and speed of neural function recovery and could improve neural function more effectively [109]. However, it has not been determined which route of administration can maximize the therapeutic effect and reduce adverse reactions.

Because of the small size and low density of EVs, they usually coexist with various particles with similar properties, resulting in a difficult separation. Several standard separation methods are used, but it remains difficult to purify the EVs. However, pre-paring exosomes using these methods usually requires longer processing times and specialized equipment.

Currently, the main technologies to protect stored EVs are freezing, freeze drying, and spray-drying [110]. The most frequently used technology, cryogenic storage, may change EV shape and physical characteristics. Repeated freeze–thaw cycles may result in changes in the biological characteristics, content, and marker composition of the EV surface molecules. However, a freeze-drying method can be used to preserve EVs using trehalose as a protective agent. Trehalose has the cryoprotective effect of stabilizing protein, cell membrane, and liposomes, preventing the aggregation of proteins and EVs, reducing ice formation during freezing, and reducing the loss of extracellular vesicles during separation and preservation [111]. Even so, more research is needed to optimize these methods and explore other approaches.

To our knowledge, there are some clinical and preclinical trials on stroke patients (shown in Table 2 and Table 3). However, a large number of clinical trials are still needed to confirm the efficacy and safety of EVs.

## 9. Conclusions

The uses of EVs for diagnostic, prognostic, and therapeutic purposes, and as drug delivery tools have been well demonstrated and continue to be a subject of intense study based on the increasing number of articles on the topic. In ischemic stroke, EVs have the function to ameliorate neurological function, reduce the permeability of BBB and brain edema, regulate inflammatory and immune response, attenuate neural apoptosis and adjust autophagy, and modulate angiogenesis and neurogenesis. As for hemorrhage stroke, there’re direct effects of EVs on stroke: anti-apoptosis, reducing inflammation, angiogenesis and immunomodulation. Additionally, it has become a trending issue to make EVs carriers for drug delivery. However, treatment with extracellular vesicles is still a nascent therapy. Many aspects remain to be resolved before being translated to the clinic as a typical practice, such as the time of administration, the most effective route, and a dose–response study. More studies are needed to evaluate the long-term biological safety, possible adverse effects, and efficacy of exosome administration in patients with stroke.

## Figures and Tables

**Table 2:** List of clinical trials on clinicaltrials.gov investigating the application of EVs for stroke.

NCT Number	Sources	Country	Conditions
NCT05370105	Circulating EVs in the serum	Italy	Stroke
NCT05524506	Microparticles	Sweden	TIA and ischemic stroke
NCT04266639	RBC-derived EVs	Denmark	Acute ischemic stroke
NCT05645081	endothelial derived EVs	UK	TIA
NCT06612710	EVs derived from neural stem cell	China	Ischemic stroke

**Table 3:** List of part of preclinical trials in recent five years investigating EVs for stroke in vivo.

Animal Model	Stroke Model	Source of EV	Methods	Key Results	Ref.
65 healthy male SD rats (8 weeks)	OGD	mesenchymal stem cells and brain endothelial cells	Ultracentrifuge	BMSC-EVs exerted similar antagonistic efficacy to BEC-EVs on Cav-1-dependent ZO-1 and Claudin-5 endocytosis.	[112]
448 adult male C57BL/6J mice (8-10 weeks)	MCAO	circSCMH1	Centrifuge	circSCMH1-EVs afford protection in the rodent and the nonhuman primate ischemic stroke models.	[113]
124 young male C57BL/6 mice (8-12 weeks)	OGD	brain endothelial cells	Ultracentrifuge	(PEG-DET/HSP27)/EV and EV/HSP27 mixtures increase BEC metabolic function and tight junction integrity.	[114]
122 ICR male mice (8-10 weeks)	MCAO	microglial BV2 cells	Ultracentrifuge	M2 microglia could communicate to OPCs through M2-EVs and promote white matter repair via miR-23a-5p after ischemic stroke.	[115]
36 male SD rat (250-300g)	MCAO/R	Que/mAb GAP43-Exo	Surface modification	Que/mAb GAP43-Exo can improve survival of neurons by inhibiting ROS production.	[116]
56 male SD rat (10 weeks)	MCAO	RBP-Exo *	Transfection engineered	RBP-Exo was a hypoxia-specific carrier for nose-to-brain delivery of AMO181a-chol in an ischemic stroke model.	[117]
60 male SD rat (8 weeks)	MCAO	BDNF-hNSC-Exo **	Coculture	BDNF-hNSC-Exo inhibited the activation of microglia and promoted the differentiation of endogenous NSCs.	[118]
45 male SD rat (8 weeks)	tMCAO	EXO-Hep ***	Ultrasonic	EXO-Hep mitigated ischemic injury in a model of tMCAO.	[119]
60 adult male C57BL/6 mice (8–10 weeks)	OGD/R	OGD/R-ADEXs	Centrifuge	Nampt released by OGD/R-ADEXs ameliorated acute ischemic stroke during neuronal injury to induce autophagy.	[120]
45 rat (8 weeks)	OGD	astrocyte-derived EVs	Ultracentrifuge	miR-29a in astrocyte-derived EVs inhibits BIRI by downregulating TP53INP1 and the NF-κB/NLRP3 axis.	[121]

* RBP: RAGE-binding-peptide; ** hNSC-Exo: human neural stem cell-derived exosomes; *** EXO-Hep: heptapeptide-loaded macrophage-derived exosomes.
